# Cardiac Fibroblast-Induced Pluripotent Stem Cell-Derived Exosomes as a Potential Therapeutic Mean for Heart Failure

**DOI:** 10.3390/ijms21197215

**Published:** 2020-09-29

**Authors:** Efrat Kurtzwald-Josefson, Naama Zeevi-Levin, Victor Rubchevsky, Neta Bechar Erdman, Orna Schwartz Rohaker, Ortal Nahum, Edith Hochhauser, Ben Ben-Avraham, Itskovitz-Eldor Joseph, Dan Aravot, Yaron D. Barac

**Affiliations:** 1 The Department of Cardiothoracic Surgery, Rabin Medical Center, Beilinson Hospital, Petah Tikva 49100, Israel; efratkur@gmail.com (E.K.-J.); victorru@clalit.org.il (V.R.); orna.rohaker@gmail.com (O.S.R.); ortalnahum@gmail.com (O.N.); aravot_dan@clalit.org.il (D.A.); 2 Technion-Israel Institute of Technology, The Ruth & Bruce Rappaport Faculty of Medicine, Haifa 3200003, Israel; naama.zeevilevin@gmail.com (N.Z.-L.); neta.bachar@gmail.com (N.B.E.); itskovitz@rambam.health.gov.il (I.-E.J.); 3 Cardiac Research Lab, Felsenstein Medical Research Center, Sackler School of Medicine, Tel Aviv University, Tel Aviv 6997801, Israel; aditho@clalit.org.il; 4 Sackler Faculty of Medicine, Tel Aviv University, Tel Aviv 6997801, Israel; bba@bgu.ac.il; 5 The Department of Cardiology, Rabin Medical Center, Beilinson Hospital, Petah Tikva 49100, Israel

**Keywords:** heart failure, iPSCs, exosome

## Abstract

The limited regenerative capacity of the injured myocardium leads to remodeling and often heart failure. Novel therapeutic approaches are essential. Induced pluripotent stem cells (iPSC) differentiated into cardiomyocytes are a potential future therapeutics. We hypothesized that organ-specific reprogramed fibroblasts may serve an advantageous source for future cardiomyocytes. Moreover, exosomes secreted from those cells may have a beneficial effect on cardiac differentiation and/or function. We compared RNA from different sources of human iPSC using chip gene expression. Protein expression was evaluated as well as exosome micro-RNA levels and their impact on embryoid bodies (EBs) differentiation. Statistical analysis identified 51 genes that were altered (*p* ≤ 0.05), and confirmed in the protein level, cardiac fibroblasts-iPSCs (CF-iPSCs) vs. dermal fibroblasts-iPSCs (DF-iPSCs). Several miRs were altered especially miR22, a key regulator of cardiac hypertrophy and remodeling. Lower expression of miR22 in CF-iPSCs vs. DF-iPSCs was observed. EBs treated with these exosomes exhibited more beating EBs *p* = 0.05. vs. control. We identify CF-iPSC and its exosomes as a potential source for cardiac recovery induction. The decrease in miR22 level points out that our CF-iPSC-exosomes are naïve of congestive heart cell memory, making them a potential biological source for future therapy for the injured heart.

## 1. Introduction

Cardiovascular diseases and their associated complications are a principal cause of morbidity and mortality worldwide [1]. The adult human heart has limited intrinsic regenerative capacity, and therefore, the myocardium lost following a myocardial infarct is typically replaced by non-contractile scar tissue, often initiating congestive heart failure (HF). Currently, the gold standard of treatment for HF patients is cardiac transplantation. Unfortunately, congestive HF patients suffer from high morbidity and mortality rates, due to shortage of donor hearts, post-transplant complications, as well as long-term failure of the transplanted hearts. Hence, novel therapeutic approaches for improving cardiac function and/or preventing HF are imperative [2].

Cell therapy is considered as one of the promising emerging technologies, i.e., transplantation of healthy, functional cells that may regenerate or repair the infarcted or ischemic myocardium [3,4]. Takahashi and Yamanaka [5] demonstrated generation of induced pluripotent stem (iPS) cells upon retrovirally transduced overexpression of four transcription factors (Oct4, Sox2, c-myc, and Klf4) in mouse dermal fibroblasts. In light of these findings, human iPS cells can currently easily be generated from human fibroblasts using either the same [6,7], or a slightly different combination of transcription factors and are considered as a potential source of stem cells with a regenerative capacity [8].

In the last decade, another novel source of cells with a regenerative capacity was identified: The cardiac stem cells population (CSC), a population that encompasses stem cell characteristics, e.g., self-renewing, clonogenic, and multipotent [9]. The CSC population are identified as they express specific membrane markers, e.g., receptor kinase c-kit [10], and were shown to be the main source of the adult myocardium regenerative capability [11]. Other studies have demonstrated that a few sub populations exist, e.g., the Lin−c-kitpos CSCs (c-kit positive cells and negative for blood/endothelial markers) that differ from other CSC and are able to differentiate not only to cardiomyocytes but also to endothelial cells and smooth muscle cells [12]. However, it has been shown that their cardio regenerative capability may be affected by different factors such as a diploid level of c-kit receptors [13], and that while they retain their cardiomyogenic potential (as can be expected from a cardiac stem cells), manipulation methods to fully use their cardio regenerative properties both in vivo and in vitro are still lacking [14].

Exosomes, nano-sized vesicles containing macromolecular substances such as proteins, lipids, and nucleic acids, serve as a central mediator in intercellular communication [15]. When first discovered, exosomes were widely regarded as cellular waste [16]; however, they have recently been shown to play an important role in many physiological and pathological processes, such as antigen presenting, tumor growth, and metastasis, as well as tissue repair [17]. Studies using exosomes from stem cells or from whole blood, in models of acute ischemia and reperfusion and chronic ischemia, have indicated that exosomes can be protective against cardiac injury [18]. In addition to decreasing the initial infarct size, they stimulate angiogenesis, reduce fibrosis and remodeling, alter immune cell function, and improve long-term cardiac contractile function.

Therefore, the present work explored the potential use of human cardiac fibroblasts (CF) or dermal fibroblasts (DF) in cardiac healing. These fibroblasts were reprogrammed into induced pluripotent stem cells (CF-iPSCs or DF-iPSCs) and exosomes secreted from these cells were collected, purified, and used as a potential therapeutic mean for cardiomyocytes development. We further focused on human cardiac iPSC, their secreted exosomes, and their potential beneficial effect on cardiac regeneration.

## 2. Results

### 2.1. Fibroblast Reprogramming

**Figure 1:**
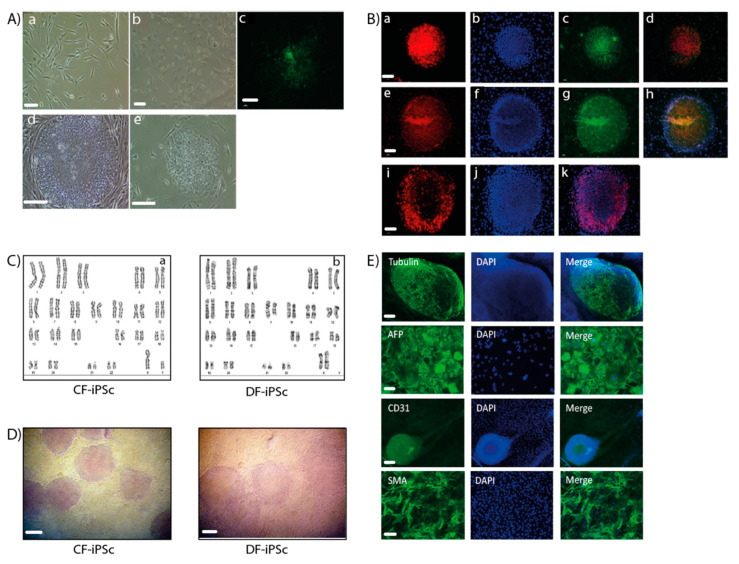
Generation of induced pluripotent stem cells (iPSCs). (**A**,**a**) A representative image of skin fibroblasts isolated from skin biopsies. (**b**) A representative image of cardiac apex fibroblasts isolated from cardiac apex biopsies. Scale bars (**a**,**b**), 100 µm. (**c**) Immunostaining of live cells using TRA-1-60 marker, derived from reprogramming the skin fibroblasts. (**d**) Representative image of a skin fibroblast-derived iPSC colony. (**e**) Representative image of a cardiac apex fibroblast-derived iPSC colony. Scale bars (**C**,**E**), 200 µm. (**B**) Immunofluorescence detection of cellular marker expression in iPSC. iPSC demonstrated staining of TRA 1–60 (**a**), Nanog (**c**), Sox2 (**e**), Oct4 (**g**), and TRA-1-81 (**i**). DAPI (4′,6-diamidino-2-phenylindole) staining of the nuclei (**b**), (**f**), and (**j**). Merged staining images (**d**), (**h**), and (**k**). Scale bars (**a**–**k**), 200 µm. (**C**) Karyotype of CF-iPSC (**a**) and DF-iPSC (**b**). (**D**) Alkaline phosphatase staining of cardiac fibroblasts (CF-iPSC)- and skin (DF-iPSC)-derived iPSCs. Scale bars, 100 µm. (**E**) iPSC lines can spontaneously differentiate into the three germ layers. Microdissected contracting areas were stained for typical myofilament proteins. Immunostaining of iPS-derived embryoid bodies (EBs) revealed the expression of ectodermal (βll-tubulin; scale bars, 200 µm), endodermal (AFP (alpha fetoprotein); scale bars, 10 µm), and mesodermal (CD31, SMA (smooth muscle actin**)**; scale bars, 20 µm) markers. Nuclei were stained with DAPI.

Ventricular and dermal fibroblasts isolated from cardiac apex and dermal biopsy samples (Figure 1A) were reprogrammed into iPSCs. CF-iPSCs and DF-iPSCs clones were propagated successfully for 30 passages, during which they maintained an ESC-like morphology (Figure 1A). iPSC’s normal karyotype was validated (Figure 1C). The pluripotency of the derived iPSCs was verified by alkaline phosphatase staining (Figure 1D) and by immunofluorescence staining, which demonstrated the expression of typical pluripotency markers Oct4, Nanog, SSEA4, TRA1-60, and TRA1-81 (Figure 1B,E).

### 2.2. Cardiomyocyte Formation

**Figure 2:**
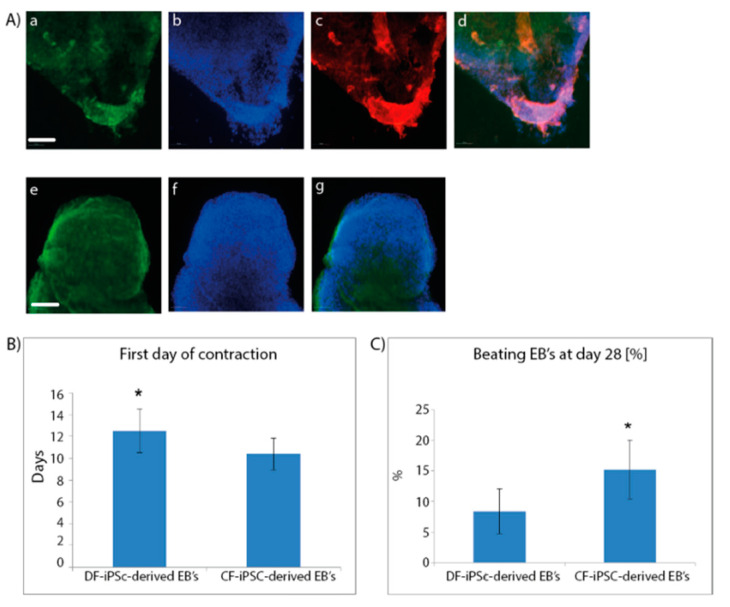
Immunofluorescence staining of cardiac proteins in iPSC-derived cardiomyocytes at day 28 of cells’ spontaneous differentiation. (**A**) Microdissected contracting areas were stained for typical myofilament proteins. Cells were labeled with antibodies specific to myosin (**a**), troponin (**c**), and α-actinin (**e**). DAPI staining of the nuclei; (**b**,**f**). Merged images; (**d**,**g**). Scale bars (**a**–**g**), 100 µm. (**B,C**) (*n* = 11). Cardiac differentiation; Plated EBs were monitored over 4 weeks of culture for (**B**) the onset of the first contracting areas (* p = 0.02). Number of contracting EBs were counted out of the total number of plated EBs ((**C**), * *p* = 0.009).

To differentiate the iPSC into the three germ layers and towards cardiomyocytes, both CF-iPSCs and DF-iPSCs were subjected to differentiation through embryoid body (EB) formation. Microdissected contracting areas were stained for typical myofilament proteins (Figure 2A). The generated EBs were monitored daily for the appearance of contracting areas, demonstrating the cardiac differentiation of the iPSCs. CF-iPSCs derived from ventricular fibroblasts demonstrated a higher tendency for cardiac differentiation, as manifested by the higher percentage of contracting EBs (Figure 2C, *p* < 0.05). In addition, CF-iPSC-derived EBs began contracting 2 days earlier than DF-iPSC-derived EBs (Figure 2B, *p* < 0.05).

### 2.3. Different Expression of Gene Cluster in CF-iPSCs vs. DF-iPSCs.

**Figure 3:**
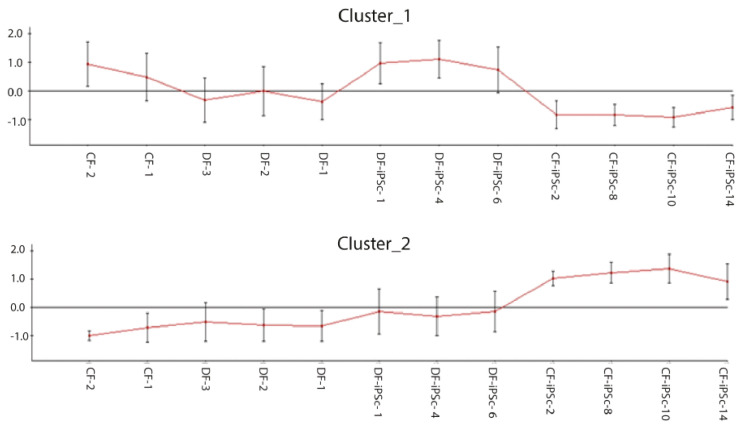
Microarray chip gene expression; 6367 differentially expressed probes selected by padj ≤ 0.05 and absolute log FC ≥ 0.75 were clustered to 11 clusters (data not shown); clustering of differentially expressed genes in CF-iPSCs versus DF-iPSCs. Cluster 1: Highly expressed in DF-iPSC vs CF-iPSC; cluster 2: Highly expressed in CF-iPSC vs DF-iPSC (*p* ≤ 0.05 and absolute log FC ≥ 0.75).

Genome-wide gene expression profiling was performed to compare the iPSCs derived from the different origins, and to determine whether some parental cell characteristics were preserved. Approximately 14,000 out of 47,000 probes analyzed were expressed in both parental fibroblast groups and both corresponding iPSC groups. Among these, 6367 probes were found to be significantly different and 11 clusters were formed (data not shown). Clusters one and two had opposite expression between CF-iPSC and DF-iPSC (Figure 3). Cluster 1: Highly expressed in DF-iPSC vs. CF-iPSC; cluster 2: Highly expressed in CF-iPSC vs DF-iPSC (*p ≤* 0.05 and absolute log FC ≥ 0.75). Contrast analysis between CF-iPSCs and DF-iPSCs gene expression identified 51 genes that were altered (Table 1). Pivotal genes relating to cardiac development and essential for cardiac mechanical and electrical contraction were further investigated in the protein level.

### 2.4. Protein Expression Levels

**Figure 4:**
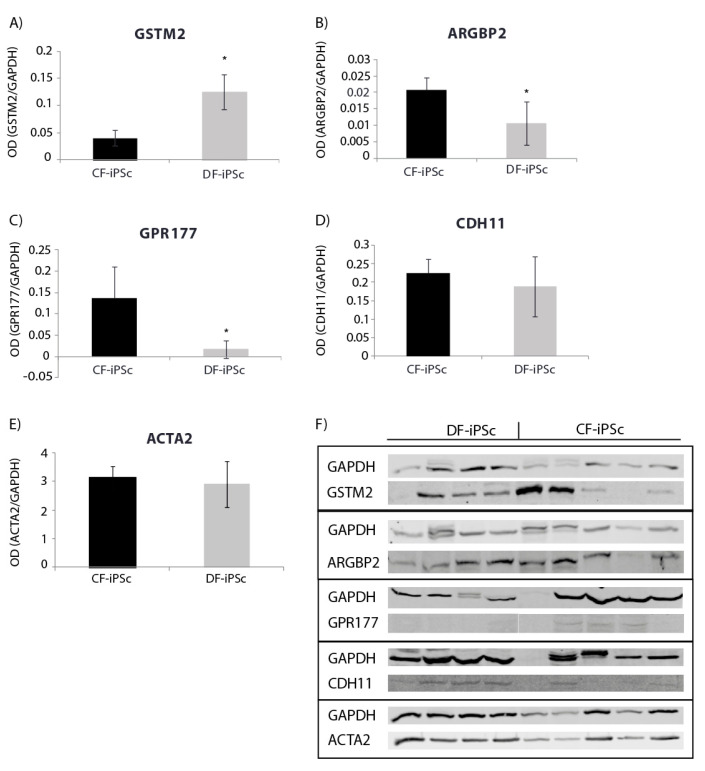
Western blot for iPSC protein (p16–18). (**A**) GSTM2 * *p* < 0.05. (**B**) ARGBP2 * *p* < 0.05. (**C**) GPR177 * *p* < 0.05. (**D**) CDH11. (**E**) ACTA2. (**F**) Blotting: CF-iPSC (*n* = 4), CF-iPSC (*n* = 5).

Protein expression levels of genes involved in cardiac development, cardiac mechanical, and electrical function were evaluated. Corresponding differences were noted at the protein expression level in CF-iPSCs and DF-iPSCs. The expression of GSTM, causing a reduction in spontaneous contractility [19], was higher in DF-iPSC, while the expression of ARGBP2, playing an important role in the assembly and maintenance of myofibrils [20,21,22], and of GPR177, playing a role in regulating Wnt secretion [23], was higher in the CF-iPSCs (Figure 4A–C, *p* < 0.05). ACTA2 (alpha smooth muscle actin) and CDH11 (Cadherin-11) protein levels showed no significant differences between the two iPSCs (Figure 4D,E).

### 2.5. Exosomes microRNA Expression

**Figure 5:**
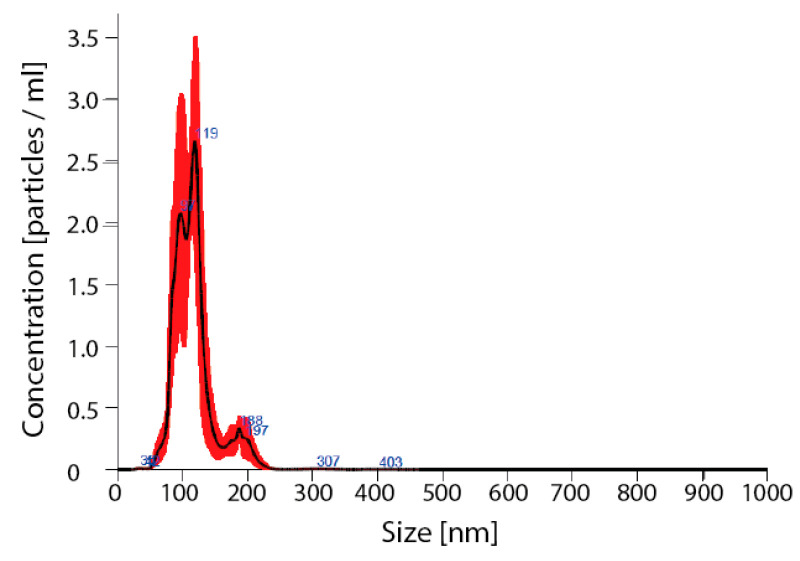
Verification of nanoparticles as exosomes. Nano sight for nanoparticles size; avg 115 ± 7, mode 111 ± 6 nm, SD 27 ± 3.

Detailed analysis of exosomes showed an average particle size of 115 ± 7, mode 111 ± 6 nm, SD 27 ± 3 (Figure 5).

**Figure 6:**
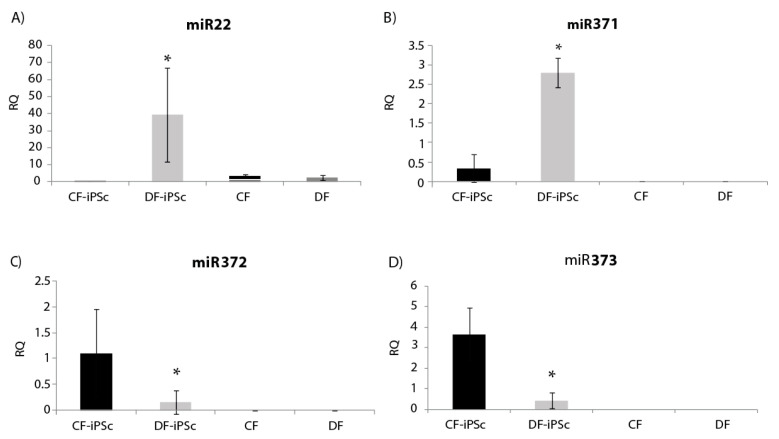
miR expression in iPSCs-derived exosomes. (**A**) miR22 expression in iPSCs exosomes, * *p* < 0.05. (**B**) miR371 expression, * *p* < 0.05. (**C**) miR372 expression, *p* < 0.05. (**D**) miR373 expression, *p* < 0.05. CF-iPSC (*n* = 5), DF-iPSC (*n* = 5), CF (*n* = 4), DF (*n* = 5).

Four microRNAs related to cardiac hypertrophy and pluripotent cells (miR22, miR371, miR372, and miR373) were analyzed in the isolated exosomes. miR22, known to be elevated in states of cardiac hypertrophy [24], showed a significantly lower expression in CF-iPSC exosomes in comparison to DF-iPSCs exosomes and to both parental fibroblasts (Figure 6A, *p* < 0.05). miR-371–373, which are prominently expressed in human embryonic stem cells (ESCs) and whom levels rapidly decrease after cell differentiation [25], were all found to be expressed in DF-iPSC and CF-iPSC exosomes but not in fibroblast exosomes (DF and CF) *p* < 0.05 (Figure 6B,D).

### 2.6. Exosome Influence on Contracting Cells

**Figure 7:**
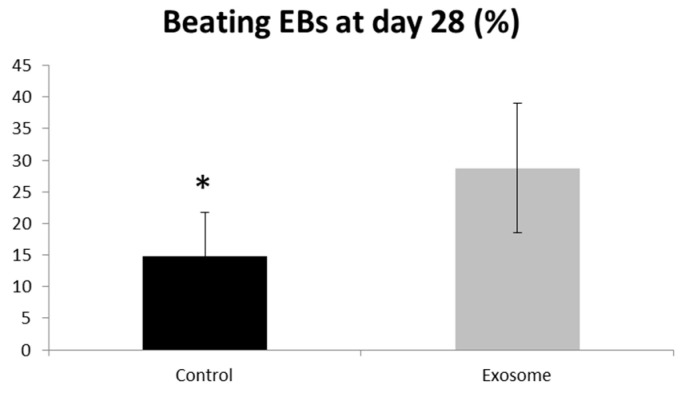
Exosome implication on beating EBs. EBs were seeded on a 6-well plate. The number of contracting EBs was calculated as a percentage of the total number of EBs. EBs treated with exosomes were compared to control,* *p* = 0.05. Control (*n* = 5), exosome (*n* = 5).

Purified CF-iPSC exosomes were used as a “cytokine-like” bio-reagent in order to induce an improved differentiation into cardiomyocyte in spontaneously differentiating EBs. Comparison between EBs treated with exosomes vs. a control group (no treatment) was performed. The percentage of beating EBs was significantly higher in the exosome-treated group, *p* = 0.05 (Figure 7).

## 3. Discussion

This study compared iPSCs derived from dermal fibroblasts vs. iPSCs derived from cardiac fibroblasts extracted from HF patients undergoing LVAD (left ventricle assist device) implantation and evaluated their potential as a source for cardiac regeneration.

The two cell sources demonstrated distinct gene and protein expression profiles; specifically, genes and proteins involved in cardiac development and cardiac mechanical and electrical contraction were altered. Furthermore, we have demonstrated that exosomes secreted from these cells have a positive impact on contracting EBs.

The first step of the study was to evaluate the differences between iPSCs derived from different origins and to determine whether and which parental cell characteristics are preserved. For that purpose, we have conducted genome-wide gene expression profiling that pointed out 51 genes that were altered (Table 1). Next, we evaluated changes in several specific cardiac-related genes in the protein level.

We found several intriguing changes that may be significant when trying to evaluate the regenerative capability of these cells. Specifically, *ARGBP2* and *GPR177* mRNA and protein levels were elevated in the cardiac iPSC lines. ARGBP2 is a 70 kDa protein which is localized to the z-bands of cardiomyocytes and plays an important role in the assembly and maintenance of myofibrils [20,21,22]. In GPR177, specification of myo- and endocardial precursor cells occurs early during the embryonic development within the mesodermal germ layer. Data gathered from studies performed in the embryonic period suggest the active participation of different growth factors; for example, the function of different Wnt factors is altered during myocardial specification. [26]. GPR177 plays a role in regulating Wnt secretion in a diverse range of tissue types [23]. The elevation in both proteins (Figure 4B,C) may contribute to the differences in the contractility timing and efficacy (evaluated by the percentage of beating EBs at the last day of the experiment—day 28) of cardiac- vs. dermal-derived EBs (Figure 2B,C).

The glutathione transferases (GST) structural family modulates ryanodine receptor (RyR) isolated from muscles (cardiac and skeletal) [27]. GSTM2 is an inhibitor of cardiac RyR2 [28,29], and it has been shown to be expressed in human skeletal and cardiac muscle [30]. Hewawasam et al. showed that exogenous treatment of neonatal cardiomyocytes with GSTM2 caused a reduction in spontaneous contractility [19]. Low protein levels of GSTM2 can be related to a normal cardiomyocyte development, with no memory of an illness (Figure 4A). Characterizing the iPSC lines through RNA and protein may explain the physiological differences (Figure 3 and Figure 4). Both *CDH11* and *ACTA2* genes were upregulated in CF-iPSC vs. DF-iPSC cells, but were identical at the protein level (Figure 4D,E). These changes in the RNA level but not in the protein level suggest that the cell morphology is the same, but it may function differently (while differentiated to EBs). CDH11 interacts with CDH2, which has been shown to interact with β-catenin. β-catenin is a subunit of the cadherin protein complex and acts as an intracellular signal transducer in the Wnt signaling pathway. Normal mesoderm formation and initiation of gastrulation in embryos was associated with Wnt signaling and the presence of β-catenin. Embryos lacking β-catenin were unable to develop mesoderm and initiate gastrulation [31]. Taken together, these differences observed between the derived iPSC types suggest that the origin of the reprogrammed somatic cells is crucial for cell function. Hence, reprogrammed cardiac fibroblasts may serve as a superior source for cardiomyocyte differentiation and may serve as an alternative for future cardiac regeneration.

Future innovative strategies for cardiac regeneration may involve nanoparticles such as exosomes. Lately, it has been demonstrated that exosomes have diverse beneficial effects on the injured heart. Milano et al. [32,33] have reported that exosomes released from human CPCs are beneficial in animal models of acute myocardial infarction or ischemia/reperfusion injury. In their latest study, the ability of human CPC exosomes to inhibit Dox/Trz-mediated oxidative stress in cardiomyocytes was presented [34]. Furthermore, in a different study, exosomes secreted from cardiac progenitor cells (CPC), which were injected in vivo to mice post-MI (myocardial infarction), induced cardiac function improvement, scar reduction, and a higher percentage of viable areas when compared to control mice [32]. Lai et al. were among the first to show that exosomes from mesenchymal stem cells (MSCs) are cardioprotective; exosomes released by MSCs in vitro were injected into the tail veins of mice undergoing myocardial ischemia and caused a reduction in infarct size 24 h later and cardiac function improvement within 28 days [35,36]. Further studies showed that in ex-vivo experiments, the MSC exosomes protected the isolated perfused heart, i.e., protection was independent of the circulating immune cells [36]. Furthermore, Wang et al. showed that exosomes isolated from mouse CF-iPSC-conditioned medium, intra-myocardially injected into mouse ischemic myocardium before reperfusion, protected against myocardial ischemia/reperfusion injury [37]; others have shown that exosomal micro-RNAs can potently alter the transcriptome of recipient cells [38]. As exosomes are highly conserved microvesicles [39], they play a central role in disease formation.

Our comparative analysis found a decrease in miR22 expression in CF-iPSC exosomes as compared to DF-iPSC exosomes and in the parental CF exosomes (Figure 6A); miR22 is known to be elevated in cardiac hypertrophy and remodeling [40]. A reduction in the expression of miR22 levels reflects on a memory loss of the “hypertrophy” diseased state. While prior studies demonstrated that CM-derived iPS generated from 1-day-old mice CMs retained an “epigenetic memory” of their “cardiogenic fate” [41], in the current study (where we showed memory loss) the CF-iPSC were generated from a different source—a human ventricle one. Therefore, we believe that our CF-iPSC and their exosomes are naïve comparing to the DF-iPSC and do not have a cell memory of a congestive heart.

The miR 371–373 cluster, typically highly expressed in ESCs and which rapidly declines after cell differentiation [25], was expressed in iPSC exosomes, attesting to their true regenerative potential (Figure 6B,D). In summary, CF-iPSCs exosomes exhibited pluripotent ability and no pathological hypertrophy, providing both beneficial primary factors, alongside loss of “pathology memory”.

Once we evaluated the effect of these exosomes on cardiac differentiation, their beneficial effect on contraction was positive and substantial (Figure 7, *p* = 0.05). This result projects on the positive physiological implication of these exosomes on the heart and perhaps on the injured heart.

This study aimed to identify a potential biological source for future therapy of the injured heart. The comparison of different origins of iPSCs demonstrated potential advantages of cardiac-derived iPSCs in attenuating cardiac remodeling. Cell therapy has its drawbacks when considering clinical trials. Nevertheless, cell products, such as secreted exosomes, may be considered as a clinical tool. We suggest that exosomes secreted from CF-iPSC will be investigated as a potential therapeutic tool in the future. Further in vivo studies are in need to establish its physiological effect and possible beneficial outcome.

## 4. Materials and Methods

### 4.1. Generation and Maintenance of Patient-Derived Human-Induced Pluripotent Stem Cells

Cardiac apex and dermal biopsies were obtained from four male patients with advanced HF who underwent left ventricle assist device (LVAD) implantation. These fibroblasts (cardiac apex and dermal biopsies) were reprogrammed into iPSCs using lentivirus or CytoTune^®^-iPS Sendai Reprogramming Kit (Life Technologies, Waltham, MA, USA), as per the manufacturer’s instructions and as previously described [42]. Briefly, human fibroblasts were plated into a 6-well plate in fibroblast medium (Biological Industries, Beit Haemek, Israel) so that they were 30–60% confluent on the day of transduction. Fibroblasts were transduced using the lentivirus or CTS™ CytoTune™ 2.1 Sendai reprogramming vectors (Life Technologies, Waltham, MA, USA) at the appropriate MOI (multiplicity of infection). Spent medium was replaced every other day. After eight days, medium was changed into complete Essential 8™ Medium (Life Technologies, Waltham, MA, USA). Spent medium was replaced every day and cell culture was monitored for the emergence of iPSC colonies. When iPSC colonies were ready for transfer (days 21–28), live staining was performed (TRA-160, Life Technologies, Waltham, MA, USA), and undifferentiated iPSCs were picked and transferred onto fresh culture dishes for expansion.

Colonies were maintained in either Dulbecco’s modified eagle’s medium (DMEM)/Nutrient Mixture F-12 Ham 1:1 (Biological Industries, Beit Haemek, Israel) containing 20% KnockOut SR (Life Technologies, Waltham, MA, USA), 1% nonessential amino acid, 1% L-glutamine (Biological Industries, Beit Haemek, Israel), 0.2% 2-mercaptoethanol (Life Technologies, Waltham, MA, USA), and rhFGF basic (4 ng/mL) (R and D Systems, Minneapolis, MN USA), or NutriStem hESC XF (Biological Industries, Beit Haemek, Israel) containing rhFGF basic (4 ng/mL), 5% human serum albumin (Biological Industries, Beit Haemek, Israel), and 1% pen-strep (Biological Industries, Beit Haemek, Israel), at 37 °C, 95% air, and 5% CO_2_ in an incubator. Derived iPSC cells were maintained either on a mouse embryonic fibroblasts (MEF) feeder layer or on Cultrex-coated tissue culture dishes (Trevigen, Gaithersburg, MD, USA). Cells were passaged using collagenase type 4 (Worthington, Columbus, OH, USA) every 3–4 days.

### 4.2. Cardiomyocyte Differentiation

Human iPSCs were grown to 80% confluence. To induce cardiomyocyte differentiation, iPSCs were dispersed into small clumps by incubation with collagenase type 4 (Worthington, Columbus, OH, USA) for 60 min, cultivated as embryoid bodies (EBs) in suspension of EB’s medium containing DMEM (Biological Industries, Beit Haemek, Israel) containing 20% FBS (Thermo Scientific, Waltham, MA, USA), 1% L-glutamine (Biological Industries, Beit Haemek, Israel), 1% pen-strep (Biological Industries, Beit Haemek, Israel), 1% nonessential amino acids, and 0.2% 2-mercaptoethanol. Cell were cultivated on 55-mm plates for 7 days, plated on 0.1% gelatin-coated culture dishes, and examined daily for the appearance of spontaneous contractions. The percentage and the onset of beating EBs were compared between CF-iPSCs and DF-iPSCs clones. Alternatively, the dispersed iPSC colonies were incubated on 55-mm plates for 14 days in EB’s medium. EBs were then treated with collagenase type B (Worthington, Columbus, OH, USA) for 30 min and seeded on a 0.1% gelatin (Sigma–Aldrich, St. Luis, MO, USA)-coated tissue culture dish for another 7 days. Spontaneous differentiation of iPSCs into cells of endoderm, mesoderm, and ectoderm lineages was then detected by immunofluorescence staining [43].

### 4.3. Immunostaining iPSc

Reprogramming efficiency was evaluated by live cell immunostaining using pluripotency marker Tra-1–60. Positive-stained colonies were picked from the plate and passaged. For each patient, several human iPSC clones were generated and continuously propagated, characterized, and used for cardiomyocyte differentiation. Colonies were seeded on 0.1% gelatin-coated culture dishes for 3–4 days. Specimens were fixed with methanol (Bio-Lab, Jerusalem, Israel) for 15–20 min, and permeabilized using 1% Triton-X-100 (Sigma–Aldrich, St. Luis, MO, USA) for 15 min, followed by blocking in 2% normal goat serum for 25 min at room temperature. The colonies were then incubated overnight at 4 °C with primary antibodies (Table 2) and then rinsed ×3 with Dulbecco’s phosphate buffered saline (DPBS 1X) (Life Technologies, Waltham, MA, USA) for 30 min. Samples were then incubated with secondary antibodies (Table 2) for 1 h, rinsed, treated with a drop of DAPI (4′,6-diamidino-2-phenylindole) fluoromount-G (SouthernBiotech, Birmingham, AL USA), and covered with a coverslip. Images were acquired using a Nikon DS-Fi2 microscope with image studio software [42].

### 4.4. EBs Characterization

EBs were immune-stained for both germ layer and cardiomyocytes biomarkers. Both CF-iPSCs and DF-iPSCs were subjected to a differentiation protocol and monitored daily for the appearance of contracting areas. Cells were labeled with βIII tubulin (ectodermal), alpha-fetoprotein (AFP) (endodermal), and CD31, smooth muscle actin (SMA) (mesodermal) markers. Microdissected contracting areas were stained for typical myofilament proteins: Myosin, α-actinin, and troponin.

### 4.5. Alkaline Phosphatase Staining

Colonies were fixed with 4% formaldehyde in DPBS for 1 min, and then rinsed with TTBS buffer 10× (Bio-Lab, Jerusalem, Israel). Alkaline phosphatase (AP) was stained using the alkaline phosphatase detection kit (Millipore, Burlington, MS, USA) as per the manufacturer’s instruction [44].

### 4.6. Microarray Chip Gene Expression Analysis

Microarray expression profiling was performed in the Genomics Core Facility (BioRap Technologies, Rappaport Research Institute, Technion, Haifa, Israel). Total RNA was harvested from three CF-iPSCs clones, four DF-iPSCs clones, and the parental DF and CF for microarray analysis. Four different cell groups were analyzed: Dermal or cardiac fibroblasts and the corresponding derived dermal or cardiac iPSCs. RNA (100 ng) was amplified into biotinylated cRNA using the TargetAmp Nano labeling kit for Illumina BeadChips, (Epicentre, Madison, WI, USA). Biotinylated cRNAs was purified, fragmented, and subsequently hybridized to an Illumina HumanHT-12 v4 Expression BeadChip using the direct hybridization assay (Illumina Inc. San Diego, CA, USA). The hybridized chip was stained with streptavidin-Cy3 (MERCK, Darmstadt, Germany) and scanned with an Illumina HiScan. The scanned images were imported into GenomeStudio (Illumina Inc. San Diego, CA, USA) for extraction and quality control.

### 4.7. Exosome Purification

Exosomes were extracted from iPSCs using total exosome isolation reagent (Thermo Fisher Scientific, Waltham, MA, USA) as per the manufacturer’s instructions. Briefly, iPSCs were cultured in 6-well plates, until 80% confluence, in serum-free iPSC growth medium containing Dulbecco’s modified eagle’s medium (DMEM)/Nutrient Mixture F-12 Ham 1:1 (Biological Industries, Beit Haemek, Israel) containing 20% KnockOut SR (Life Technologies, Waltham, MA, USA), 1% nonessential amino acid, 1% L-glutamine (Biological Industries, Beit Haemek, Israel), 0.2% 2-mercaptoethanol (Life Technologies, Waltham, MA, USA), and rhFGF basic (4 ng/mL) (R and D Systems, Minneapolis, MN, USA), at 37 °C, 95% air, and 5% CO_2_ in an incubator. Culture medium was then collected and centrifuged at 2000× *g* for 30 min. The supernatant was then transferred to a new tube and mixed well with 5 mL of total exosome isolation reagent. The homogenous solution was incubated at 4 °C overnight and then centrifuged at 4 °C at 10,000× *g* for 1 h. The supernatant was then aspirated, and the exosomes were suspended in 500 µL PBS and stored at −80 °C until use. Extracted exosomes were analyzed for particle visualization and rapid, automated analysis of size distribution and concentration by nanoparticle tracking system (NanoSight NS300, Weizmann institution, Rehovot, Israel). Exosomes were saved at −80 °C for following experiments.

### 4.8. Exosome Implication on EBs

EBs were cultured as described above (cardiomyocyte differentiation). Exosome treatment began at day 7. Exosome were applied (5 × 10^8^ ± 1 × 10^8^ particles) every other day for 10 days and compared to control wells (no treatment). The amount of contracted EBs were taken at the last day of the experiment. Percentage of contracted EBs (exosome-treated compared to the control) were calculated.

### 4.9. Micro-RNA Extraction and RT-PCR

Total exosomal RNA was extracted using a Total Exosome RNA and Protein Isolation Kit (Thermo Fisher Scientific, Waltham, MA, USA) in accordance with the protocol recommended by the manufacture (enrichment for small RNA). The quantity of microRNA was determined by NanoDrop^TM^ Spectrophotometer. cDNA was synthesized from microRNA using the TaqMan High-Capacity cDNA Reverse Transcription Kit (Applied Biosystems; Foster City, CA, USA) in accordance with the manufacturer’s protocol; only specific primers were used and not random ones. Reverse transcription was performed as follows: 30 sec at 16 °C, 30 sec at 42 °C, 5 min at 85 °C and 4 °C. Quantitative real-time PCR analysis was performed using the ABI 7000 Sequence Detection System (Applied Biosystems, Foster City, CA, USA). The primers and TaqMan FAM were synthesized by Applied Biosystems (Foster City, CA, USA). A total of 1 µL cDNA was amplified with 5 µL TaqMan Fast Advanced Master MIX, 0.5 µL specific primers for U6 (Thermo Fisher Scientific, Waltham, MA, USA), miR22 (Thermo Fisher Scientific, Waltham, MA, USA), or miR210 (Thermo Fisher Scientific, Waltham, MA, USA) and 3.5 µL DEPC. PCR amplification was performed using the following program: 2 min at 50 °C, 20 sec at 95 °C, and 40 cycles of 20 sec at 60 °C. Human U6 was used as a housekeeping gene. Quantitative values were obtained by cycle threshold (CT) values. Relative mean fold changes were calculated using the 2^−∆CT^ method.

### 4.10. Protein Extraction and Western Blot Analysis

Proteins were extracted from cells using CytoBuster reagent (MERCK, Darmstadt, Germany), as per the manufacturer’s instructions. Briefly, iPSCs were cultured in 6-well plates, until reaching 80% confluence, in NutriStem hESC XF (Biological Industries, Beit Haemek, Israel) at 37 °C, 95% air, and 5% CO_2_ in an incubator. Culture medium was collected and centrifuged at 800 rpm for 3 min. The supernatant was aspirated, and the cells were mixed well with CytoBuster and then centrifuged at 16,000× *g* for 5 min. The supernatant was transferred to a new tube, and stored at −80 °C until use. Proteins (50 μg/sample) were separated on a sodium dodecyl sulfate (SDS) polyacrylamide gel (12%) under denaturing conditions and electro-transferred onto a nitrocellulose membrane (Bio-Rad, Hercules, CA, USA), over 1 h at 100 V. Membranes were blocked with 5% nonfat milk in Tris–buffered saline with 0.1% Tween 20 (TBST), for 1 h at room temperature. Membranes were then incubated with the following antibodies, diluted in TBST with 5% BSA, overnight, at 4 °C: anti-ARGBP2 ((Thermo Fisher Scientific, Waltham, MA, USA), anti-CDH11 (Thermo Fisher Scientific, Waltham, MA, USA), anti-GPR177 (Thermo Fisher Scientific, Waltham, MA, USA), anti-GSTM2 (Thermo Fisher Scientific, Waltham, MA, USA). Anti-GAPDH (Thermo Fisher Scientific, Waltham, MA, USA), was performed as an internal control. After rinsing, membranes were incubated with dye 800/680-labeled secondary antibodies (1:10,000) for 1 h, at room temperature, as well as donkey anti-mouse IRDye 680, donkey anti-goat IRDye 680, and donkey anti-rabbit IRDye 800 (LI-COR Biosciences, Lincoln, NE, USA). Immunofluorescence was detected with the Li CORE Odyssey (LI-COR Biosciences, Lincoln, NE, USA) and signals were quantified with the Image Studio Lite Ver 5.2 program.

### 4.11. Ethics Statement

All protocols were approved by the Rabin Medical Center Helsinki Committee, 0502-12-RMC 6/30/2013- 6/28/2021. Informed consent was acquired from HF patients for collection of skin and cardiac apex tissue in order to isolate fibroblasts and generate induced pluripotent stem cells (iPSC).

### 4.12. Statistical Analysis

Downstream analysis of microarray gene expression was performed at the INCPM Weizmann Institute using Expander (clustering with click) and Ingenuity IPA. Results are reported as mean + SEM. *p* < 0.05 was considered statistically significant. Unpaired t-test or two-way ANOVA were used to assess statistical significance between groups. A statistical significance was followed by post-hoc comparisons.

## Figures and Tables

**Table 1:** Contrast analysis—genes that were altered.

CF-iPSC > DF-iPSC	DF-iPSC > CF-iPSC
*ACTA2*	*AGTPBP1*
*CDH11*	*CEBPZ*
*GPR177*	*CLDN7*
*PALLD*	*CYP2S1*
*KIAA1514*	*HS574598*
*SORBS2*	*HS575676*
*SORBS2*	*LOC143272*
*TYW3*	*MT1H*
*ACTN3*	*PRDM14*
*ANKRD32*	*PRKCQ*
*CRYZ*	*SCGB3A2*
*G3BP2*	*UCA1*
*HSZ142*	*XIST*
*HSPC157*	*C17ORF11*
*HSPC157*	*C21ORF11*
*LOC100128775*	*CIDEB*
*CYORF15A*	*LOC311796*
*DNAJC15*	*LOC729774*
*EIF1AY*	*MRPL41*
*EIF1AY*	*NANOG*
*JARID1D*	*RPL22L1*
*LOC100133662*	*UCKL1*
*NLGN4Y*	*GSTM2*
*OPNYSW*	
*RPS4Y1*	
*RPS4Y2*	
*SMC1*	
*TTTY15*	

**Table 2:** Lineage marker-specific antibodies.

Name	Source	Dilution
**Primary antibodies**		
Anti-Oct4	Millipore	1:100
Anti-Sox2	Millipore	1:100
Anti-TRA-1-60	Millipore	1:100
Anti-Tra-1–81	Millipore	1:100
Anti-Nanog	Millipore	1:100
Anti-CD31	BioLegend	8 µg/mL
Anti-AFP	Abnova	30 µg/mL
Anti-actin smooth muscle	Millipore	1:100
Anti-neuronal class III β-Tubulin (TUJ1)	BioLegend	1:1000
Anti-human alpha actinin	Millipore	1:250
Anti-myosin	Millipore	1:10
Anti-cardiac troponin I	Abcam	1:100
**Secondary antibodie**	Jackson	
Alexa Fluor 488-conjugated AffiniPure donkey anti-mouse IgG	ImmunoResearch Jackson	1:500
Cy3-conjugated AffiniPure donkey anti-rabbit igG	ImmunoResearch	1:500

## Abbreviations

iPSC	induced pluripotent stem cells
EBs	embryoid bodies
CF	cardiac fibroblasts
DF	dermal fibroblasts
HF	heart failure
ESCs	embryonic stem cells
RyR	ryanodine receptor
MSCs	mesenchymal stem cells
MEF	mouse embryonic fibroblasts
